# Multimorbidity patterns in dementia and mild cognitive impairment

**DOI:** 10.3389/fpsyt.2024.1432848

**Published:** 2024-11-07

**Authors:** José Alejandro Valdevila Figueira, Rocío Valdevila Santiesteban, Indira Dayana Carvajal Parra, Luis Patricio Benenaula Vargas, Andrés Ramírez, Jose E. Leon-Rojas, Jose A. Rodas

**Affiliations:** ^1^ Faculty of Marketing and Communication, Universidad Ecotec, Guayaquil, Ecuador; ^2^ Research Network in Psychology and Psychiatry (GIPSI), Guayaquil, Ecuador; ^3^ Institute of Neurosciences, Junta de Beneficencia de Guayaquil, Guayaquil, Ecuador; ^4^ Carrera de Psicología Clínica, Universidad Politécnica Salesiana, Cuenca, Ecuador; ^5^ Escuela de Medicina, Universidad de las Américas, Quito, Ecuador; ^6^ Escuela de Psicología, Universidad Espíritu Santo, Samborondón, Ecuador; ^7^ School of Psychology, University College Dublin, Dublin, Ireland

**Keywords:** dementia, mild cognitive impairment, medical comorbidity, diagnosis and classification, latent class analyses

## Abstract

**Design:**

This is a retrospective cohort study. Setting: The study was conducted at the Instituto de Neurociencias de la Junta de Beneficencia de Guayaquil, a primary neuroscience institute in Ecuador.

**Participants:**

The study evaluated 425 participants diagnosed with Mild Cognitive Impairment (MCI) or dementia, out of which 272 individuals (mean age = 75 years; 164 female) presenting specific medical conditions were selected for analysis.

**Measurements:**

Data were collected on demographics, medical history, and neuropsychological assessment using the Neuropsi scale. Conditions such as Type 2 Diabetes Mellitus, hypertension, obesity, and history of traumatic brain injury were specifically noted.

**Results:**

Latent Class Analysis identified three distinct classes of patients: Unspecified Cognitive Deterioration, Dementia, and MCI. The three-class model provided the best fit, revealing varied morbidity patterns and highlighting the influence of vascular and metabolic conditions on cognitive decline. Notably, similarities in hypertension and diabetes prevalence between Dementia and MCI classes suggested shared risk factors. The study also found no significant age differences between the classes, indicating that age alone might not be the primary determinant in the progression of cognitive decline.

**Conclusions:**

The study underscores the complexity of dementia and MCI in an ageing Ecuadorian population, with vascular health playing a crucial role in cognitive impairment. These findings advocate for a holistic approach in managing dementia and MCI, emphasising the importance of addressing cardiovascular and metabolic health alongside neurocognitive care. The distinct morbidity patterns identified offer insights into tailored intervention strategies, highlighting the need for comprehensive, multidisciplinary care in dementia management.

## Introduction

Dementia is a syndrome characterised by the decline of cognitive functions and interpersonal relationships, marked by mood changes, memory loss, difficulties in acquiring new knowledge, and, more importantly, an inability to carry out activities of daily living (1). The prevalence of dementia varies globally, with rapid growth observed in Latin America and the Caribbean, where women face a 65% higher risk of experiencing disability-adjusted life years (DALYs) compared to the global average of 60%. Approximately 10 million individuals in the Americas currently live with dementia (2), with Alzheimer’s Disease (AD) being the predominant cause, accounting for 60 to 80% of cases (3).

Globally, around 50 million people are affected by dementia, with projections suggesting an increase to 75 million by 2030 and up to 135 million by 2050 (4–6). This condition, prevalent in 7% to 14% of individuals over the age of 65, leads to significant disability and places a substantial burden on caregivers due to neuronal degeneration that results in cognitive, functional, emotional, and social decline (7). Moreover, individuals with mild cognitive impairment (MCI) are at a significantly higher risk of progressing to AD, further impairing daily functioning and increasing the likelihood of additional health complications (8).

Ecuador’s demographic changes provide a clear example of the challenges presented by an ageing population, which directly correlates with the rising incidence of dementia. In 1950, Ecuador had an average life expectancy of 53.2 years at birth, with 41.1% of the population under 15 years of age. Over the next four decades, life expectancy increased to nearly 73 years, accompanied by steady population growth and negligible differences in lifespan across genders. Since the turn of the millennium, life expectancy has continued to rise at a rate of 1 year for every 5 years lived, and the demographic over 60 years of age, which barely surpassed half a million two decades ago, now exceeds 2 million (9).

Currently in Ecuador, dementia poses a significant health challenge, with an evenly distributed male and female population. Currently, 8% of the population is over 65 years old (10). Data from the Ecuadorian Institute of Statistics indicate a decline in birth rates alongside an increase in life expectancy by five years each decade. This trend towards greater longevity, coupled with reduced birth rates, implies that Ecuador will experience a significantly aging population in the near future, potentially leading to a rise in the prevalence of dementia and other neuropsychiatric disorders (11).

Providing appropriate emotional care to individuals with dementia is becoming an increasingly significant challenge worldwide. The quality of life and functional status of those living with dementia are profoundly influenced by the quality of care they receive across a diverse range of health settings. This diversity necessitates a multitude of strategies to effectively manage comorbidities associated with dementia (12).

Due to the complexity and variety of symptoms and complications inherent in dementia, alongside the comorbidities that develop over time, delivering effective medical care is particularly challenging. Individuals with dementia frequently require comprehensive, multidisciplinary care that spans mental, physical, and social services. The nature of care provided plays a crucial role in determining the quality of life and overall well-being of the patient, underscoring the necessity to enhance treatment approaches as both a social and medical priority. Factors such as Type 2 Diabetes Mellitus, high blood pressure, obesity, advanced age, varying levels of education, and a history of repetitive mild brain injuries contribute to approximately 35% of dementia cases today (13).

Recent research highlights the crucial impact of comorbidities and underlying factors on the progression and outcomes of dementia and MCI. These findings are essential for developing targeted interventions and managing the overall health of those affected by these conditions. The incidence of medical comorbidities in individuals with MCI is particularly high, with conditions such as hypertension, diabetes, cardiovascular diseases, and depression being widespread (11, 14, 15).

For example, Stephan et al. (14) discovered that more than half of the individuals in each cognitive group suffered from at least one medical condition, with hypertension being the most common (11). Moreover, the Mayo Clinic Study of Aging (14), revealed considerable variability in mortality rates among MCI subtypes, influenced by sex, lifestyle choices, and comorbidities, further emphasising the significance of these factors on the health outcomes of individuals with MCI.

The link between multimorbidity and the increased risk of developing dementia, including AD and vascular dementia, has been highlighted by several studies (i.e., 16–18) In the study by Hu et al. (17), a dose-response relationship between the number of long-term health conditions and the risk of dementia was found, indicating that individuals with four or more chronic conditions faced a significantly higher risk.

Veronese et al. (18), in their analysis of the SHARE cohort, revealed that multimorbidity significantly increased the risk of dementia, particularly among younger individuals, with those aged 55 or younger facing over double the risk. The study also identified specific comorbidities such as high cholesterol, stroke, diabetes, and osteoporosis as key contributors to dementia risk, especially in those aged 60 to 70. Calvin et al. (16) further supported these findings, demonstrating that multimorbidity was associated with a 63% increased risk of dementia, with disease clusters involving cardiovascular and metabolic conditions, such as hypertension, diabetes, and coronary heart disease, posing the highest risks.

The impact of illness in AD and its precursor, MCI, extends beyond cognitive decline to include a broad spectrum of comorbidities such as cardiovascular diseases and diabetes, prevalent among affected individuals (17). A systematic review by Lanctôt et al. (19) underlines the high prevalence of these conditions, stressing the need for comprehensive health management for individuals with AD and MCI (17). Moreover, mortality rates among those with MCI and a high cardiovascular risk further highlight the crucial connection between cardiovascular health and cognitive function. Research by Yaneva-Sirakova and Traykov (20) points to hypertension as a primary risk factor, with MCI patients suffering from hypertension showing a significantly higher mortality rate, thus emphasising the importance of early detection and management of cardiovascular risks in this demographic (19).

In light of the evolving definitions of AD, the study by James and Bennett (21) draws attention to the multifactorial essence of dementia. It acknowledges the significance of mixed pathologies as well as the roles of neural reserve and resilience. This approach affirms the complexity of dementia and advocates for a comprehensive strategy in prevention and treatment that addresses both pathological and non-pathological factors (20). Furthermore, multimorbidity may not present randomly but often follows specific patterns. For instance, the study by Vassilaki et al. (15) revealed that a combination of cardiovascular, metabolic, and depressive disorders is particularly harmful, increasing the risk of developing dementia. In addition, Marengoni et al. (22) expanded on this by demonstrating that distinct multimorbidity patterns also contribute to disability in older adults. Their six-year longitudinal study of older individuals in Sweden found that specific multimorbidity clusters, such as cardiovascular/anaemia/dementia and musculoskeletal/respiratory/gastrointestinal, were strongly associated with a higher risk of disability in both basic and instrumental activities of daily living. These findings suggest that certain multimorbidity patterns not only raise the risk of cognitive decline but also significantly impair functional abilities, further complicating the management of dementia and disability in ageing populations. Recognising these patterns is crucial for developing targeted prevention strategies and optimising healthcare planning to delay both cognitive and functional impairment.

The present study aims to elucidate the patterns of morbidity and cognitive profiles among patients diagnosed with dementia and MCI through Latent Class Analysis (LCA). The study seeks to categorise these individuals into distinct classes based on their cognitive performance and diagnostic characteristics. This classification allows for a deeper understanding of the varied manifestations of cognitive decline and their underlying health profiles, providing insights into the common and divergent paths of disease progression within this population.

## Methods

### Participants

All participants were inpatients and outpatients from the Instituto de Neurociencias de la Junta de Beneficencias de Guayaquil (Neuroscience Institute from the Guayaquil Charity Board) seen between 2010 and 2022. 425 participants with a diagnose of MCI or dementia were evaluated with the Neuropsi scale (21), however, only 272 (mean age = 75 years old, min = 60, max = 97; 164 female) also presented one of the following medical conditions: osteoartritis, diabetes, traumatic brain injury, hyperlypidemia, epilepsia, alcoholism, Parkinson disease, hypothyroidism, high blood pressure or obesity. Participants with a history of drug use were excluded. Results from the analysis using the Neuropsy will be reported elsewhere. Most inpatients were diagnosed with dementia, while the outpatients could present both dementia or MCI.

### Instruments

In this study, we employed two primary instruments to assess the patterns of multimorbidity in dementia and MCI: the Neuropsi and the Clinical History. The Neuropsi is a comprehensive neuropsychological test battery, validated and adapted for Spanish-speaking populations, which evaluates various cognitive functions, such as participants’ (a) orientation to time, place, and self, alongside (b) attention and concentration through tasks like digit span, visual detection, and mental arithmetic. Memory assessment focuses on both (c) encoding, through verbal word learning and visuospatial figure copying tasks, and (d) recall by asking participants to retrieve the learned words and reproduce the copied figure after a delay. Language (e) tasks measure naming, repetition, comprehension, and verbal fluency. Executive functions (f) are tested through problem-solving, sequencing, and motor coordination, while (g) reading and (h) writing tasks evaluates literacy skills (23). This test is particularly valuable for detecting cognitive impairments related to dementia and other neurological disorders, as it assesses abilities such as word recall, story and figure retention over time, and logical reasoning. For this study, we used a total score calculated from all sub-scales to capture an overall measure of cognitive performance.

Additionally, the Clinical History provided a detailed medical record of each participant, including personal and family history, the presence of physical and mental health conditions, as well as previous and current treatments. Dementia was diagnosed when a participant experienced significant cognitive decline in one or more domains, such as memory, language, or executive function, and this decline interfered with daily functioning and could not be attributed to any other medical condition. In the case of MCI, this decline was also present but did not interfere with daily activities.

### Procedure

The data were collected from the follow-up medical records of subjects previously diagnosed with dementia or cognitive impairment, stored in the Archives and Statistics Department of the INC. A database was created in Excel to facilitate analysis and enhance understanding. The search focused on identifying pathologies mentioned during the preparation of the clinical history, including Type 2 Diabetes Mellitus (DM-2), High Blood Pressure (HBP), epilepsy, hypothyroidism, hyperlipidaemia (confirmed through laboratory data), obesity, osteoarthritis, the history of traumatic brain injury, alcoholism, and Parkinson’s Disease (PD).

### Statistical analysis

All statistical analyses in this study were performed using jamovi v2.4.11 (24), built on R (25). This analysis incorporated the poLCA (26) and snowRMM (26) packages. The initial step involved presenting descriptive statistics from our sample, focusing on age and cognitive functioning comparisons using Analysis of Variance (ANOVA). Our primary method, Latent Class Analysis (LCA), was employed to discern distinct profiles or patterns of multimorbidity among participants. Unlike methods that rely on predefined categories, LCA allows classes to emerge directly from the data, grouping individuals based on shared characteristics, in this case, patterns of comorbid conditions. This unsupervised approach enables the discovery of latent subgroups within the sample, revealing unique morbidity profiles that may not have been anticipated beforehand.

To ascertain model fit, we computed various indices: the Akaike Information Criterion (AIC), Bayesian Information Criterion (BIC), sample-size adjusted BIC (SABIC), and consistent AIC (CAIC). In these indices, lower values suggest a more robust data fit. Recognising the diverse diagnoses in our participants, we controlled for cognitive functioning impacts in our morbidity pattern analysis by incorporating the Neuropsi score as a covariate in the LCA.

### Ethical considerations

As this investigation posed no risk, clinical records were reviewed. The data were handled anonymously and with confidentiality. The study complied with the principles of the Declaration of Helsinki (1964) and adhered to the stipulations of the Political Constitution of the Republic of Ecuador (27). Approval was secured from the INC’s Teaching and Research Department and the centre’s technical management.

## Results

### Sample characteristics

**Table 1** illustrates the sample’s diagnostic characteristics. Predominantly, participants had some type of dementia, with Unspecified Dementia being most common. Age comparisons showed no significant differences (F (6, 265) = 1.72, p = .117). Contrastingly, significant cognitive functioning variations were noted when assessed with the Neuropsi total score (F (6, 265) = 8.45, p <.001, η² = .161). *Post hoc* analyses revealed notable differences between various dementia types and Mild Cognitive Impairment (MCI), corrected for multiple comparisons via the Tukey procedure.

**Table 1:** Frequencies of diagnosis.

Diagnosis	N	% of Total	Cumulative %	Mean Age (SD)	Mean Neuropsi score (SD)
Dementia in Alzheimer’s Disease	63	23.2 %	23.2 %	75.6 (7.21)	42.8 (26)
Dementia in Parkinson’s Disease	7	2.6 %	25.7 %	74 (8.1)	52.9 (25.2)
Dementia in Pick’s Disease	3	1.1 %	26.8 %	70 (5.57)	40 (25.5)
Unspecified Dementia	83	30.5 %	57.4 %	76.2 (6.46)	51 (25)
Vascular Dementia	52	19.1 %	76.5 %	75.5 (7.16)	51.4 (20.4)
Dementias in Other Specified Diseases Classified Elsewhere	3	1.1 %	77.6 %	75 (6.56)	44.3 (22.7)
Mild Cognitive Impairment	61	22.4 %	100.0 %	72.9 (7.65)	71.5 (21.9)

SD, standard deviation.

### Morbidity pattern identification

**Figure 1:**
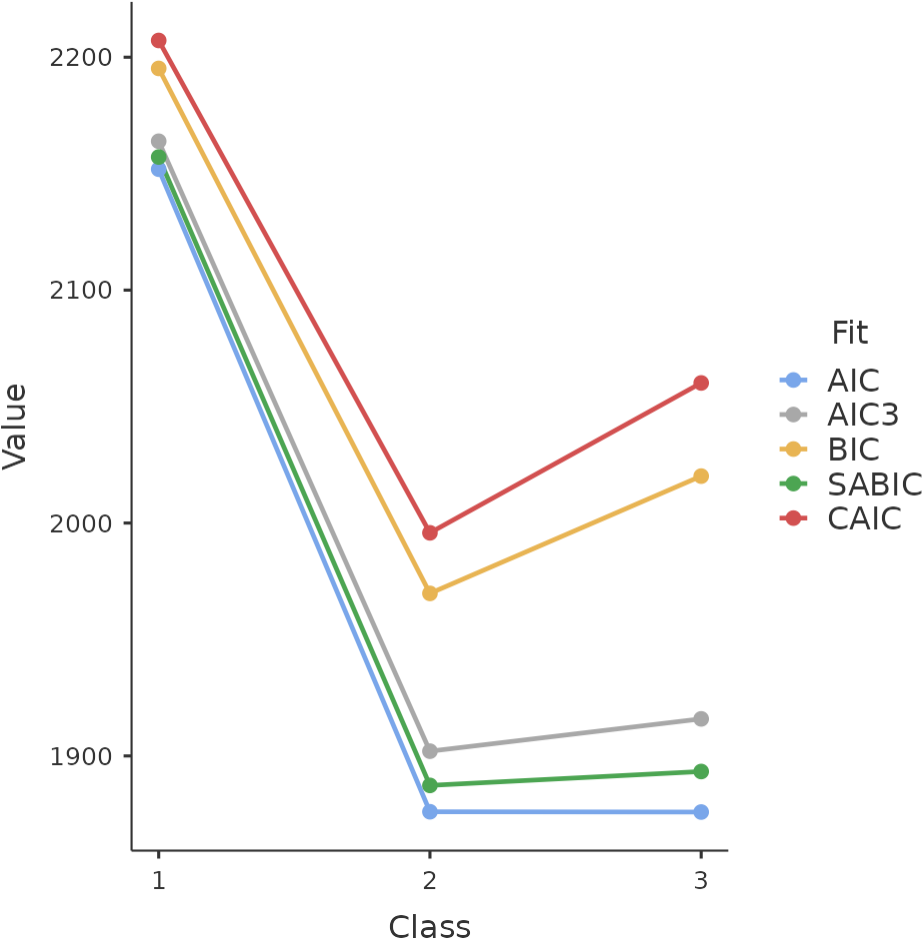
Elbow plot depicting fit indices from four models.

LCA was conducted on a sample of 272 participants, encompassing both dementia and MCI patients. In these analyses, we used the total score from the Neuropsi cognitive evaluation as a control variable across all models. The findings revealed distinct morbidity patterns within the patient group. Among four tested models, the three-class model demonstrated the most suitable fit for a majority of the indices, as illustrated in **Figure 1**. This model exhibited an entropy value of 0.89, indicative of a good fit (28).

While the BIC and CAIC lent support to the two-class models, which distinguished participants solely into MCI or dementia categories, the AIC, AIC3, and SABIC favoured the three-class model. This more complex model provides an expanded understanding of morbidity patterns, going beyond the basic MCI-dementia dichotomy of the two-class models.

**Figure 2:**
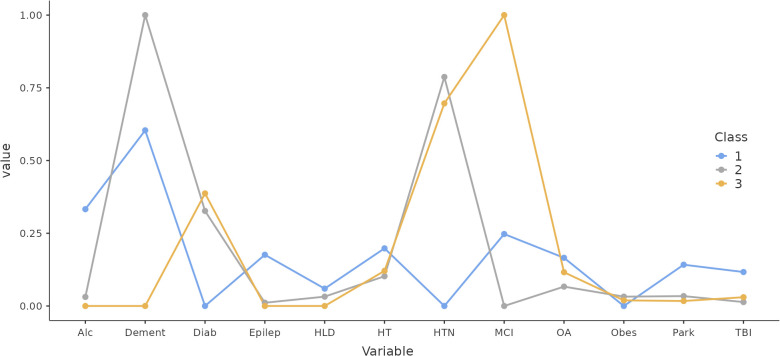
Profile Plot from the Three Classes. Alc, alcoholism; Dement, dementia; Diab, diabetes; Epilep, epilepsia; HLD, hyperlipidaemia; HT, hypothyroidism; HTN, hypertension; MCI, mild cogni-tive impairment; OA, osteoarthritis; Obes, obesity; Park, Parkinson disease; TBI, traumatic brain injury.

**Figure 2** displays three distinct patterns discernible amongst the classes. Class one, characterised as Unspecified Cognitive Deterioration, comprises 12.4% of the sample. Class two, identified as Dementia, encompasses 68.8% of the sample, and class three, representing MCI, includes 19% of the sample. The profile of class one shows elevated probabilities of conditions such as alcoholism, epilepsy, hypothyroidism, Parkinson’s disease, and traumatic brain injury. Notably, while these conditions are predominantly associated with dementia, they also pose a significant risk for MCI.

An intriguing observation is the similarity in the health profiles of the Dementia (class two) and MCI (class three) classes, with only minor differences in conditions like hypertension, osteoarthritis, hyperlipidaemia, and diabetes. Both Dementia and MCI classes demonstrate a heightened risk of hypertension (approximately 75%) and diabetes (around 30%) compared to the Unspecified Cognitive Deterioration class. Logistic regression analysis further revealed the Neuropsi score’s significant influence as a control variable in this context (coefficient = -0.033, t = -3.72, p <.001).

### Differences between classes

The analysis of variance revealed no significant age differences between the classes (F (2, 269) = 2.89, p = 0.057). However, as observed from the logistic regression coefficient, the Neuropsy score varied significantly across classes (F (2, 269) = 24.2, p <.001). Notable differences were identified between class 1 (Unspecified Cognitive Deterioration) and class 2 (Dementia), with a t-score of 4.09 (p <.001, effect size d = .754), and between class 2 and class 3 (Mild Cognitive Impairment), with a t-score of -6.29 (p <.001, effect size d = -.987). These comparisons were adjusted for multiple comparisons using the Tukey procedure to ensure statistical rigour. **Table 2** provides a detailed breakdown of the descriptive statistics for each class. Furthermore, the Supplementary materials include a table that presents the number of patients for each ICD-10 diagnosis, categorised by class membership. This additional table provides insight into how the diagnoses are distributed across the different classes.

**Table 2:** Descriptive statistics from the three classes.

	Class	N	Mean	SD
Neuropsy Score	1	35	64.7	21.04
	2	185	46.9	24.57
	3	52	70.2	21.92
Age	1	35	74.1	7.22
	2	185	75.7	7.03
	3	52	73.2	7.24

## Discussion

The results of this study offer significant insights into the morbidity patterns and cognitive profiles among patients with dementia and MCI. Our LCA identified three distinct classes: Unspecified Cognitive Deterioration, Dementia, and MCI. This classification enhances our understanding of the varied manifestations of cognitive decline, their possible sources and aids in tailoring interventions more effectively.

The observed similarities in health profiles between the Dementia and MCI classes, particularly in the context of hypertension and diabetes, are particularly noteworthy. This resemblance suggests that these conditions might share common underlying risk factors or pathways leading to cognitive decline, underscoring the necessity for a more nuanced understanding and approach in both diagnosis and treatment.

While the vascular contributions to dementia are well established in the literature, our findings contribute to a deeper understanding by identifying specific comorbidity patterns involving cardiovascular and metabolic conditions that influence both dementia and MCI. By employing LCA, we uncovered distinct morbidity profiles, highlighting the critical role of conditions like hypertension and diabetes, which were prevalent across both dementia and MCI groups and are well-known risk factors for vascular diseases (29–31). Both conditions have been increasingly implicated in the development and progression of cognitive disorders. The subtle differences in hypertension and diabetes prevalence among these classes hint at the possibility of vascular contributions to their pathogenesis. This suggests that shared vascular and metabolic pathways may contribute to cognitive decline, offering new insights into the heterogeneity of cognitive impairments and the overlapping risk factors that might accelerate the progression of the disease. Moreover, our study advances the field by incorporating detailed cognitive assessments (Neuropsi), allowing for a better understanding of how specific comorbidity patterns interact with cognitive domains like memory, attention, and executive functioning, which is less commonly explored in prior LCA studies (32, 33).

In addition to its focus on an underrepresented Latin American population, our study’s use of LCA provides a novel perspective on the heterogeneity within dementia and MCI populations. The identification of latent subgroups that reflect varying comorbidity profiles contributes to a more comprehensive understanding of how chronic conditions co-occur and affect cognitive outcomes, extending beyond typical descriptive approaches. This research not only strengthens the applicability of our findings across diverse populations but also underscores the importance of addressing vascular health in the clinical management of dementia and MCI. Given the demographic and socioeconomic challenges of the population studied, our findings highlight the need for targeted interventions focusing on cardiovascular health to mitigate cognitive decline, as well as the importance of preventive care and early detection in resource-limited settings (34, 35).

Emerging research supports this link between cardiovascular health and cognitive function (36–38), suggesting that vascular health could play a crucial role in the onset and progression of cognitive impairment. Poor vascular health, as evidenced by conditions like hypertension and diabetes, may lead to reduced cerebral blood flow, contributing to the accumulation of brain pathologies and the subsequent development of cognitive decline. This insight has significant implications for the clinical management of Dementia and MCI. It suggests that a holistic approach, addressing not only neurocognitive aspects but also vascular health, might be more effective in managing these conditions. Moreover, these findings highlight the importance of early identification and management of vascular risk factors as a potential strategy for preventing or delaying the onset of cognitive decline.

The Unspecified Cognitive Deterioration class, as identified in our study, presents a unique profile within the spectrum of cognitive impairments. This class, comprising a smaller portion of our sample (12.4%), is characterised by a broader range of cognitive deficits compared to the more specific profiles of Dementia and MCI. One of the key findings regarding this class is its more unspecific health profile. Unlike the Dementia and MCI classes, which showed higher risks of vascular-related conditions like hypertension and diabetes, the Unspecified Cognitive Deterioration class did not exhibit these heightened risks. This suggests that the cognitive decline in this group may be less influenced by vascular factors and more by other, and more varied, underlying causes.

Interestingly, this class showed higher probabilities of conditions such as alcoholism, epilepsy, hypothyroidism, Parkinson’s disease, and traumatic brain injury. These conditions are known to affect brain health directly, either through neurodegenerative processes, metabolic dysfunctions, or physical trauma to the brain. This pattern indicates that cognitive decline in this class might be predominantly driven by these direct neurological impacts rather than secondary effects of systemic conditions like hypertension or diabetes.

The presence of diverse underlying conditions in the Unspecified Cognitive Deterioration class also suggests a more complex and multifaceted approach to diagnosis and management. The variation in potential causes for cognitive decline necessitates a comprehensive assessment strategy that goes beyond typical dementia screenings. It highlights the need for individualised diagnostic processes, considering the wide range of potential contributing factors. Furthermore, the treatment and management strategies for this class would likely differ from those for typical Dementia or MCI. Given the diversity of underlying conditions, a multidisciplinary approach to care is essential. This approach might include not only neurocognitive therapies but also targeted treatments for the co-occurring conditions like epilepsy or hypothyroidism, and lifestyle interventions for factors like alcoholism.

The lack of significant age differences as a moderator in our study, particularly in the context of cognitive impairment classes, provides an interesting perspective on the relationship between age and cognitive decline. Commonly, age is considered a crucial factor in cognitive disorders, with the assumption that older individuals are more prone to severe conditions like dementia. However, our findings challenge this assumption and offer an alternative understanding in cases where comorbidity exist.

The absence of significant age differences between the classes, especially between class 2 (Dementia) and class 3 (MCI), suggests that age may not be the primary determinant in the progression of cognitive decline when comorbidity exist. This is particularly relevant considering the traditional view that MCI often serves as a precursor to dementia, predominantly as a function of advancing age. Our results indicate that the transition from MCI to dementia might not be linearly related to ageing in cases of comorbidity but could be influenced by a complex interplay of other factors.

This observation raises important questions about the underlying mechanisms of cognitive decline. If age is not the primary driver, then other factors – be it genetic, lifestyle, environmental, or a combination of these – might play a more significant role. For instance, the similar risk profiles for conditions like hypertension and diabetes in both Dementia and MCI classes point to shared vascular risk factors that might contribute to cognitive decline irrespective of age.

Furthermore, the finding that age is not a significant moderator in the differentiation between classes has implications for how we approach the diagnosis and treatment of cognitive disorders. It suggests a need for a more nuanced approach that goes beyond age as a primary criterion. Clinicians may need to focus more on symptomatic presentation, cognitive assessment scores, and possibly genetic and lifestyle factors when diagnosing and managing cognitive decline. Additionally, this finding could influence public health strategies and awareness campaigns. It highlights the importance of cognitive health across all age groups and underscores the need for early intervention and prevention strategies that are not solely focused on the elderly.

The significant impact of the Neuropsi score as a moderator in distinguishing the classes in our study is a pivotal finding. The Neuropsi, as a comprehensive cognitive evaluation tool, played a crucial role in elucidating the subtle differences and similarities across the identified classes of cognitive impairment. Firstly, the varying Neuropsi scores across the classes underscore the heterogeneity in cognitive functioning within the spectrum of cognitive disorders. In the Unspecified Cognitive Deterioration class, for instance, the Neuropsi score might reflect a broader range of cognitive deficits, consistent with the diverse health conditions observed in this group. In contrast, the Dementia and MCI classes, while showing some overlap in vascular-related risk factors, presented distinct cognitive profiles. This suggests that despite some commonalities in underlying risk factors, the manifestation and severity of cognitive impairment differ between these classes.

### Limitations

Firstly, the population analysed was exclusively from the Institute of Neurosciences in Guayaquil and the analyses would benefit of a larger sample size. This may limit the representativeness of our findings, as this sample may not fully capture the diversity and heterogeneity of the broader Ecuadorian population. Consequently, the results might not be entirely generalisable to the entire population of the country. Secondly, the study’s focus was solely on individuals diagnosed with dementia or MCI, excluding those without these conditions. This selection criteria could affect the interpretation and applicability of our findings, as the absence of a comparative group makes it challenging to thoroughly assess the prevalence and nature of comorbidities in the population.

These limitations highlight the necessity for future research that incorporates a more diverse and representative sample. Such research should include comparisons with individuals without cognitive impairment or dementia. This approach will enable a more comprehensive and contextualised understanding of comorbidities associated with dementia and MCI.

### Final thoughts

In conclusion, our study, conducted in Ecuador—a region with limited research on cognitive disorders—provides valuable insights into the complex nature of dementia, MCI, and Unspecified Cognitive Deterioration. The identification of distinct classes with varying health profiles, particularly highlighting the possible role of vascular factors in two of these classes, is a significant contribution to the field, especially in a context where such data is scarce. These findings underscore the potential influence of general health on cognitive impairment, suggesting that conditions like hypertension and diabetes may play a more critical role than previously acknowledged.

These findings have important implications for clinical practice and public health strategies. It indicates a need for holistic assessment and intervention strategies that encompass both neurocognitive and vascular aspects. Such an approach is crucial not only for accurate diagnosis and effective treatment but also for the development of targeted prevention programs. Given the diversity of health profiles observed, especially in the Unspecified Cognitive Deterioration class, our results also advocate for more personalised care plans that address the specific needs of each patient.

Furthermore, the significance of these findings in a region with limited research highlights the need for continued and expanded studies in similar contexts. Future research should aim to include broader and more diverse populations, enhancing the generalisability of the results. This is particularly important in understanding the full spectrum of cognitive disorders in different cultural and geographical settings.

In essence, our study paves the way for a better understanding of cognitive impairments, emphasising the importance of vascular health in cognitive decline and advocating for comprehensive, individualised care approaches. It also calls for more extensive research in underrepresented regions, contributing to a more global and inclusive understanding of cognitive disorders.

## Data Availability

The raw data supporting the conclusions of this article will be made available by the authors, without undue reservation.
