# Supplementary material for: Multimorbidity patterns in dementia and mild cognitive impairment
Source: Front Psychiatry. 2024 Nov 7;15:1432848. doi: 10.3389/fpsyt.2024.1432848 (PMC11578943; doi:10.3389/fpsyt.2024.1432848)
Supplement: Supplementary file 1 [file Table1.docx]

**Supplementary materials**

| Frequencies of CIE-10 diagnoses split by membership | | | | | | | | | |
| --- | --- | --- | --- | --- | --- | --- | --- | --- | --- |
| CIE-10 | | Membership | | Counts | | % of Total | | Cumulative % | |
| Dementia in Alzheimer's Disease |  | 1 |  | 6 |  | 2.2 % |  | 2.2 % |  |
|  |  | 2 |  | 57 |  | 21.0 % |  | 23.2 % |  |
|  |  | 3 |  | 0 |  | 0.0 % |  | 23.2 % |  |
| Dementia in Parkinson's Disease |  | 1 |  | 3 |  | 1.1 % |  | 24.3 % |  |
|  |  | 2 |  | 4 |  | 1.5 % |  | 25.7 % |  |
|  |  | 3 |  | 0 |  | 0.0 % |  | 25.7 % |  |
| Dementia in Pick's Disease |  | 1 |  | 1 |  | 0.4 % |  | 26.1 % |  |
|  |  | 2 |  | 2 |  | 0.7 % |  | 26.8 % |  |
|  |  | 3 |  | 0 |  | 0.0 % |  | 26.8 % |  |
| Unspecified Dementia |  | 1 |  | 6 |  | 2.2 % |  | 29.0 % |  |
|  |  | 2 |  | 77 |  | 28.3 % |  | 57.4 % |  |
|  |  | 3 |  | 0 |  | 0.0 % |  | 57.4 % |  |
| Vascular Dementia |  | 1 |  | 8 |  | 2.9 % |  | 60.3 % |  |
|  |  | 2 |  | 44 |  | 16.2 % |  | 76.5 % |  |
|  |  | 3 |  | 0 |  | 0.0 % |  | 76.5 % |  |
| Dementias in Other Specified Diseases Classified Elsewhere |  | 1 |  | 2 |  | 0.7 % |  | 77.2 % |  |
|  |  | 2 |  | 1 |  | 0.4 % |  | 77.6 % |  |
|  |  | 3 |  | 0 |  | 0.0 % |  | 77.6 % |  |
| Mild Cognitive Impairment |  | 1 |  | 9 |  | 3.3 % |  | 80.9 % |  |
|  |  | 2 |  | 0 |  | 0.0 % |  | 80.9 % |  |
|  |  | 3 |  | 52 |  | 19.1 % |  | 100.0 % |  |
|  | | | | | | | | | |
